# A VSV-G Pseudotyped Last Generation Lentiviral Vector Mediates High Level and Persistent Gene Transfer in Models of Airway Epithelium *In Vitro* and *In Vivo*

**DOI:** 10.3390/v2081577

**Published:** 2010-08-02

**Authors:** Elena Copreni, Lucia Palmieri, Stefano Castellani, Massimo Conese

**Affiliations:** 1 Institute for the Experimental Treatment of Cystic Fibrosis, H.S. Raffaele, via Olgettina 58, 20132 Milan, Italy; E-Mails: copreni@marionegri.it (E.C.); lpalmieri@rpsweb.com (L.P.); stekas1977@hotmail.com (S.C.); 2 Department of Biomedical Sciences, University of Foggia, 71122 Foggia, Italy

**Keywords:** lentivirus, cystic fibrosis, GFP, luciferase, lysophosphatidylcholine, airway epithelial cells, lung, mice

## Abstract

The aim of this work was to evaluate the efficiency and duration of gene expression mediated by a VSV-G pseudotyped last generation lentiviral (LV) vector. We studied LV efficiency in *ex-vivo* models of respiratory epithelial cells, obtained from bronchial biopsies and nasal polyps, by GFP epifluorescence and cytofluorimetry. *In vivo* efficiency and persistence of gene expression was investigated by GFP immunohistochemistry and luciferase activity in lung cryosections and homogenates, respectively, upon intranasal and intratracheal administration protocols in C57Bl/6 mice. Both primary bronchial and nasal epithelial cells were transduced up to 70–80% 72 hr after the LV infection. *In vivo* nasal luciferase expression was increased by lysophosphatidylcholine pre-treatment of the nose. Conversely, the bronchial epithelium was transduced in the absence of any pre-conditioning treatment and luciferase expression lasted for at least 6 months without any decline. We conclude that a last generation LV vector is a promising gene transfer agent in the target organ of genetic and acquired lung diseases, as in the case of cystic fibrosis.

## 1. Introduction

Cystic Fibrosis (CF) is the most common life-shortening autosomal recessive disorder in Caucasian populations and its clinical symptoms are the consequence of mutations in the CF transmembrane conductance regulator (CFTR) gene on chromosome 7. CF is a chronic disease which requires gene delivery systems ensuring persistence of trangene expression. Early phase clinical trials have demonstrated that adenoviral and adeno-associated viral vectors could not achieve sustained CFTR expression and obtain clinical benefit [1]. HIV-1 derived lentiviral (LV) vectors are integrating vectors and considered a promising approach for CF gene therapy since they not only integrate in the host genome and ensure long-lasting expression of the therapeutic protein but also transduce quiescent cells and have been proved to be high efficient in the murine airway tract and achieve correction of the electrophysiological defect in the nose of CF mice [2]. Most of the studies on HIV-1-derived lentiviral mediated gene transfer in the airway epithelium refer to gene delivery mediated by LV vectors pseudotyped with vesicular stomatitis virus glycoprotein G (VSV-G) which is useful not only to allow virus particle concentration, but also to modulate virus interaction with the host immune system and to broaden its host range. However, VSV-G LV vectors have been shown to be inefficient in *in vivo* gene transfer into a fully differentiated epithelium [3–6] unless disruption of the epithelial barrier integrity obtained with calcium-chelating agents like EGTA [6], inhalation exposure to sulfur dioxide [3], or modification of the barrier function of the airway epithelium with lysophosphatidylcholine (LPC) [2] is obtained.

In this study we evaluated the efficiency of a HIV-1-derived last generation LV vector in *ex-vivo* and *in vivo* models of airway epithelium: primary bronchial and nasal respiratory epithelial cells, and the murine airway epithelium. Our results show an unpredictable high transduction of the distal respiratory epithelium *in vivo* in the absence of preconditioning and persistence of gene expression.

## 2. Results and Discussion

**Figure 1.**
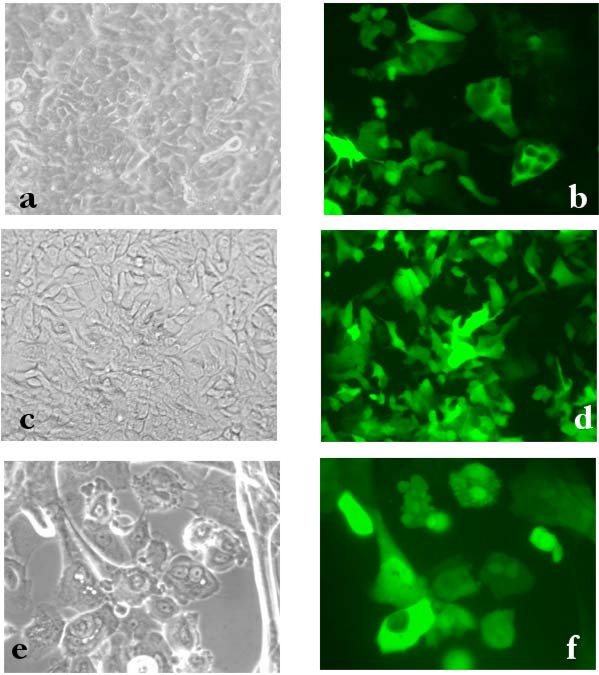
GFP expression in human bronchial and nasal epithelial cells following LV infection. Bronchial cells were infected with 100 (**a, b**) or 500 (**c, d**) MOI. Nasal cells were infected with 500 MOI (**e, f**). GFP expression was evaluated 72 hr later. **(a, c, e)** Bright fields. **(b, d, f)** Epifluorescence of the same fields. Original magnification: 40X (**a, b, c, d**); 63X (**e, f**).

In our laboratory, primary cultures of respiratory epithelial cells from bronchial biopsies and nasal polyps have been established [7–9]. Primary cells were transduced with MOIs (indicating viral particle:cell ratios) of 100 and 500. Confluent bronchial epithelial cells expressed GFP in a dose-dependent fashion (Figure 1a–d). Cytofluorimetric analysis showed that the percentage of GFP-positive cells was 54.4±4.4 and 82.5±2%, with 100 and 500 MOI respectively. Nasal epithelial cells grew more sparsely than bronchial cells, but showed high levels of GFP expression with LV infection at 500 MOI (Figure 1e and f). The percentage of GFP-positive cells was 80.3±0.1% as evaluated by cytofluorimetry. Overall, primary human respiratory epithelial cells demonstrated to be transduced *ex-vivo* by a last generation LV vector.

Transduction of the airway epithelium by VSV-G psudotyped LV vectors is not efficient because of the viral receptors (not identified yet) located on the basolateral surfaces of epithelial cells [10]. The LV vector used in this study is superior in efficiency as compared to prior versions in different models of primary cells [11]. Furthermore, we have previously shown that this LV vector is able to transduce polarized bronchial epithelial cells from the apical side, with an efficiency up to 65% [12]. Thus, the high transduction rates obtained with the last generation LV vector in primary human airway epithelial cells was promising for obtaining a good transduction of the respiratory epithelium *in vivo*. We preliminarly reported that the VSV-G pseudotyped LV vector used in this study transduced the murine bronchial/bronchiolar epithelium following an intratracheal injection [13]. To corroborate and deepen these data we studied the transduction of the airway epithelium in the respiratory tract of the mouse by comparing two delivery methods, *i.e.* intranasal *vs.* intratracheal instillation.

**Figure 2.**
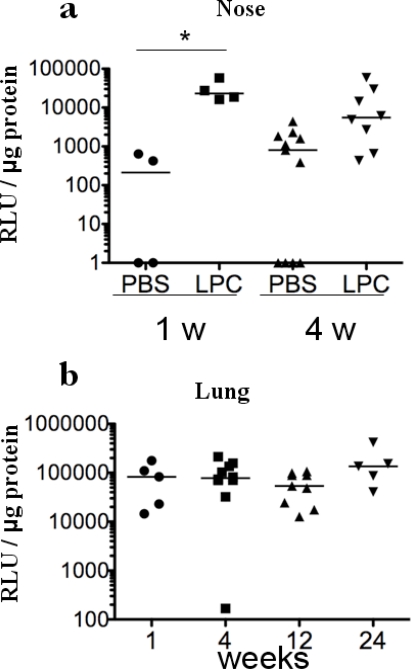
Luciferase expression in the murine airways following intranasal (**a**) or intratracheal (**b**) injection. (**a**) 1% LPC in PBS or PBS were instilled drop-wise in one nostril. One hour after, mice received a dose of 1×10^7^ TU in 20 μl of PBS of the LV-Luc vector that was administered drop-wise to the same nostril. Mice were sacrificed one week (1 w) and four weeks (4 w) after gene transfer. The nose was dissected, homogenized, and evaluated for luciferase expression. *n* = 4–11 mice per group. (**b**) The LV-luc vector was administered to the lung via the trachea and mice were sacrificed 1, 4, 12, and 24 weeks after injection. The lung was removed, homogenized, and evaluated for luciferase expression*. n* = 5–10 mice per group. *p = 0.02 (unpaired Student’s t-test).

Transduction of nasal epithelial cells by LV vectors is not achieved unless preconditioning of the upper airways is performed [2,14,15]. Since the LV vector was found to be very efficient in primary nasal epithelial cells (Figure 1e and f), the potential to transduce the nasal epithelium was attempted in the absence of any prior treatment. Luciferase activity in the nose homogenates was at the background value 1 week after instillation of 1x10^7^ transducing units (TU) (Figure 2a). We then used LPC, a naturally occurring airway surfactant that has previously been shown to increase *in vivo* airway gene transduction in the nose [2,16]. In noses pre-treated with 1% LPC, the level of luciferase activity measured one week after gene delivery was 3.0±2.3×10^4^ Relative Light Units (RLU) per microgram of protein (Figure 2a). These luciferase levels differed significantly from those achieved in the absence of pre-treatment. One month post-instillation, luciferase activity was 1.1±0.8 ×10^3^ and 1.4±0.5×10^4^ in PBS- and LPC-pre-treated animals, respectively (Figure 2a). Although a clear difference is noted, this did not reach statistical significance, due to high luciferase levels reached in 7 out of 11 of PBS-pre-treated mice. These results indicate that VSV-G LV mediated gene transfer in the nasal epithelium is enhanced by LPC pre-treatment, ensuring persistence of gene expression at least for one month post infection. Previous studies have shown that LPC preconditioning allows a VSV-G pseudotyped LV vector to transduce the respiratory epithelium, as well as the transitional epithelium [16,17].

**Figure 3.**
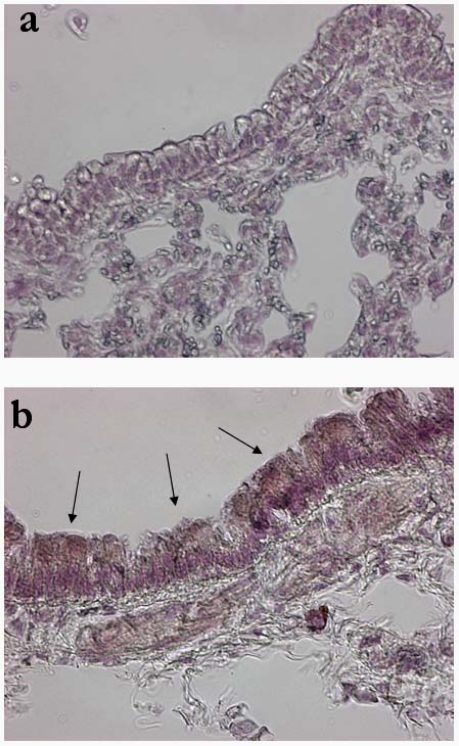
GFP expression in bronchial epithelium of mice infected with the LV vector. PBS (**a**) or the LV-GFP vector (**b**) was injected in the lung of C57BL/6 mice through an intratracheal instillation. GFP expression was evaluated 1 month later by immunohistochemistry. Black arrows indicate areas of transduced airway epithelial cells. Note the absence of staining in the PBS-injected mice. Pictures are representative of three mice per group. Original magnification: X40.

Since the target lung anatomical district for CF gene therapy is represented by the lower respiratory tract, an intratracheal injection protocol already in use in our laboratory for nonviral vectors [9,18] was used for LV application to the lung. A dose of lentivirus corresponding to 1×10^7^ TU was injected in the trachea of C57BL/6 mice and different time points after LV administration mice were sacrificed and lungs removed. GFP immunohistochemistry revealed that transduction was achieved in the bronchial epithelial cells at 1 month post-injection (Figure 3). Microscopic observation of sections stained with hematoxylin and eosin did not reveal any sign of tissue damage in LV treated lungs (not shown).

To evaluate long-term transduction of the respiratory epithelium in the lung, we injected 1×10^7^ TU of a LV vector expressing firefly luciferase. Measurement of luciferase activity in lung homogenates showed levels of 8.2±2.9×10^4^ and 9.3±1.9×10^4^ RLU per microgram of lung protein, one week and one month after gene delivery, respectively (Figure 2b). High levels of luciferase expression persisted for three and six months post injection, with measurements of 5.4±5.1 x10^4^ and 1.6±1.3×10^5^ RLU per microgram of lung protein, respectively.

In the present study we have demonstrated that a last generation VSV-G pseudotyped LV vector can mediate efficient and sustained gene expression in the mouse airways. This result is concordant with the high efficiency gained in polarized airway epithelial cells previously obtained by our group [12] and in primary cultures of human respiratory cells (this study). However, the high efficient and persistent transduction of the murine distal airways was at odds with some previous findings. Others have reported that the nasal epithelium needs to be pre-conditioned before applying the LV vector [2,16,17]. Although VSV-G pseudotyped vectors are unable to transduce the airway epithelium in differentiated xenograft model [4] and *in vivo* when administered via tracheal injection [3,6], in other studies VSV-G pseudotyped HIV vectors can transduce the alveolar epithelium [19,20] and conducting airway epithelium [21,22] in the absence of tight-junction disruption agents. The LV vector that we have used here has some advantages over the previous versions of HIV-1 lentiviral vectors, since two sequences, the PPT (PolyPurine Tract) and the WPRE (Woodchuck Post-Transcriprional Regulatory Elements) have been added to the viral genome to improve nuclear translocation and transduction efficiency [11]. Differences in LV efficiency in the lung and nose could be explained by different retention time in the two compartments [23]. Alternatively, differences in distribution of viral receptors or in intracellular processing of viral particles could account for different rate of transduction observed in the murine bronchial and nasal epithelium. Also the modality of virus administration could influence transduction outcomes, since intratracheal injection could generate a pressure in the lung, leading to the transient modification of the cellular permeability.

In this study we have shown that the LV-mediated transduction *in* vivo lasts for at least 6 months by luciferase expression. Since the whole lung is assayed, it could be that most of the luciferase expression is from transduced alveolar cells, which vastly outnumber airway epithelial cells. This notion is given credence by the work of Borok *et al.* [24], which suggested that alveolar cells are apically much more amenable to VSV-G-LV transduction than airway epithelial cells. It is thus possible that only alveolar expression persists for 6 months since the bronchial epithelium expression was not examined any later than the 4-week timepoint.

In a recent work [17], a VSV-G pseudotyped LV carrying LacZ or CFTR gene was delivered in the nostrils of mice 1 hour after pre-treatment with 0.3% LPC via inhalation-driven instillation. LacZ transgene expression lasted over 24 months and was restricted to the anterior regions of transitional and respiratory epithelium within the dosed nasal airway affecting primarily ciliated and nonciliated cells, but also a low number of secretory and basal cells. At 24 months when LacZ gene expression was still present, the number of cells transduced was significantly reduced compared to the initial 1-week level. Significant correction of CFTR function, measuring nasal potential, was present at 1 and 12 months compared to untreated mice. Although these findings are consistent with an approximate 3-month cell turnover time originally established for murine tracheal airway [25], recent data suggest longer turnover times (up to 17 months) in deeper regions of the mouse lung [26].Thus, it is currently unclear, if prolonged expression is due to vector integration into the pulmonary stem or progenitor cells or due to the long life-expectancy of airway epithelial cells. It is possible that gene expression is maintained during time despite its lowering due to cell turnover and nonetheless persistence in a stem/progenitor niche due to a transient augmentation of paracellular permeability and allowance of LV vector to deeper cell layers. The development of this vector and its derivatives in the therapy of CF lung disease merit further research in its interaction with receptors and post-entry block mechanisms in basal cells and other progenitor cells of the respiratory epithelium.

Detailed studies are necessary to address whether lentivirus-based vectors can be considered immunologically safe for gene therapy of CF. Limberis and colleagues [19] have demonstrated that transient expression of Green Fluorescent Protein (GFP) at day 90 in alveolar epithelium following an intratracheal injection of VSV-G-pseudotyped HIV-1-derived vector to the mouse lung is due to transgene- and *gag*-specific T-cell activation. However, preliminary results obtained by our group indicate that this last generation LV vector does not elicit a pro-inflammatory response in human respiratory epithelial cells *in vitro* [27]. Furthermore, we have observed that the LV vector does not induce a strong immune response in the lung, as demonstrated by absence of CD4+ and CD8+ lymphocyte infiltrates in the bronchi/bronchioli of mice intratracheally injected [28].

## 3. Experimental Section

*Production of lentiviral vectors* - The self-inactivating pRRLsin.cPPT.CMV.GFP.Wpre and pRRL.sin.PPT.CMV.luciferase.iresEMCVwtGFP.pre constructs (generously donated by Luigi Naldini, TIGET, H.S. Raffaele, Milan, Italy) were used to generate vesicular stomatitis virus glycoprotein (VSV-G)-pseudotyped LV vector stocks (LV-GFP and LV-luc, respectively) as described [11,29]. The yield of the concentrated virus was typically 10^8^–10^10^ TU/ml and the specific activity ranged between 1.56 and 4.17×10^5^ TU/ng of p24 Gag.

*Epithelial cell culture* - Primary respiratory epithelial cells were derived by enzymatic digestion with type XIV Protease (Sigma Chemical, St. Louis, MO) from bronchial specimens obtained from surgical resection, as previously described [9]. Bronchial cells were seeded on rat tail collagen and cultured in RPMI/LHC9 (1:1), 2 mM L-glutamine, 100 U/ml penicillin and 100 μg/ml streptomycin.

Respiratory epithelial cells were cultured from nasal polyps, as already described [7,8]. Briefly, explants (1–2 mm^2^ in size) of human nasal polyps were seeded onto 60-mm tissue culture dishes coated with type I collagen associated with carbodiimide (Sigma, St. Louis, MO). Explants were then incubated in serum-free RPMI-1640 culture medium in the presence of 2 mM l-glutamine, 1 mg/ml insulin, 1 mg/ml transferrin, 10 ng/ml epidermal growth factor, 0.5 mg/ml hydrocortisone, 10 ng/ml retinoic acid, 100 U/ml penicillin, and 100 μg/ml streptomycin. Epithelial cells obtained from outgrowths of nasal polyps and bronchial specimens were trypsinized and cells were plated on 24-well plates at the density of 100,000 cells/well in 1 ml of culture medium.

*Transduction of epithelial cells* - Cells were incubated with the vector in complete medium for 24 hours containing the LV particles. The lentivirus was removed, cells washed with PBS, incubated in complete medium and evaluated for GFP expression after 48 hours by epifluorescence using a Zeiss Axiovert 135 microscope equipped with GFP filter (excitation 395 nm, emission 509 nm). Cytofluorimetry was performed with a FACScan apparatus (Becton-Dickinson, San Jose, CA) at 525 nm. The percentage of GFP-positive cells was determined after setting the gating on 99% of an untransfected control population of cells and by substracting the fluorescence of the untransfected control cells. Ten thousand cells were examined in each experiment. Analysis of GFP production was performed by plotting the GFP channel (FL1) against the FL3 channel. Data are shown as the mean and standard deviations obtained from three experiments each for bronchial and nasal cells.

*In vivo experiments* - All animals procedures were conformed to the requirements of the H. S. Raffaele Institutional Animal Care and Use Committee (IACUC). Male C57BL/6 mice (26–28 gr of weight) were obtained from Charles River, Calco, Italy. For intranasal injection, mice were anesthetized with 12 mg/g body weight of Avertin and kept upright by hanging them by their teeth. Four μl of a solution of 1% LPC in PBS or PBS were instilled drop-wise in the nostril. Fourty-five minutes after, mice received 12 mg/g body weight of Avertin and a 15 minutes later a dose of 1×10^7^ TU of the LV-luc vector in 20 μl of PBS was administered drop-wise to the same nostril. Mice were sacrificed one week and one month after gene transfer. Nose was dissected and homogenized with a glass mortar and pestel in 1.5 ml of lysis buffer (25 mM Tris\HCl, 2 mM DTT, 2 mM EDTA, 10% Glycerol, 1% Triton X-100, pH = 8) on ice, homogenates were frozen in liquid nitrogen and thawed on ice and centrifuged at 12000 ×*g* at 4 °C for 20 min. Fifty μl of the supernatant were diluted with 100 μl of water and assayed for luciferase activity in a Lumat LB 9507 instrument (Berthold, Bad Wildbach, Germany) by adding 100 μl luciferase reagent (Promega) to each sample and measuring the light emitted over a 30 s period. Protein content of each sample was determined by a standard Lowry modified assay (Pierce) and the results expressed as relative light units (RLU) per μg protein. Noses from mice injected with PBS only gave consistently those which are considered background levels (< 200 RLU/μg protein).

The LV vector was administered to the lung via the trachea as previously described [9]. The vector was administered through the catheter with a Hamilton syringe at the dose of 1×10^7^ TU in 100 μl of PBS. Control mice were injected with 100 μl of PBS. Mice were sacrificed by cervical dislocation one weeks, one month, three and six months after transduction. For GFP immunodetection, lungs were removed and fixed in 4% paraformaldehyde and frozen; cryosections were stained with an antibody raised against GFP, as previously described [30]. Sections were observed at the Zeiss Axioplan-2 epifluorescent microscope. To determine the luciferase activity, the lung was dissected out, washed in ice-cold PBS and immediately stored in 10 ml of ice-cold PBS. The homogenization of the samples was performed in 1 ml of lysis buffer (25 mM Tris\HCl, 2 mM DTT, 2 mM EDTA, 10% Glycerol, 1% Triton X-100, pH = 8) using an Ultraturrax Homogenizer at the maximum speed for 40 s. Samples were frozen in liquid nitrogen, allowed to thaw on ice and then centrifuged at 12000 ×*g* at 4 °C for 20 min. Luciferase activity was determined as described for the nose. Lungs of mice injected with PBS gave consistently luciferase background levels.

## 4. Conclusions

The transduction of basal cells or other progenitor cells in the airway epithelium with VSV-G pseudotyped LV vectors warrants further studies. A VSV-G pseudotyped last generation lentiviral vector can hold promise for long-term gene transfer into the respiratory epithelium and gene therapy of chronic lung diseases, such as cystic fibrosis.
